# Research on the Modification Process of Jute Fiber as a Strengthening Material for the Structure of Solidification Substrate

**DOI:** 10.3390/ma18050937

**Published:** 2025-02-21

**Authors:** Ronglin Zhou, Wanlai Zhou, Qi Bai, Juncheng Liu, Zhiyong Qi

**Affiliations:** 1 School of Mechanical Engineering, Chengdu University, Chengdu 610106, China; zhouronglin@cdu.edu.cn (R.Z.); baiq_99@163.com (Q.B.); ljceng@icloud.com (J.L.); 2 Institute of Urban Agriculture, Chinese Academy of Agricultural Sciences, Chengdu 610213, China; zhouwanlai@caas.cn

**Keywords:** solidification substrate, jute fiber, soilless culture, three-dimensional greening

## Abstract

Substrate is the key material of soilless culture. The physical and chemical properties of the solidified cultivation medium are good and relatively stable, and there is no need to use plastic cultivation containers in the cultivation process, which has a broad application prospect in three-dimensional greening and fruit and vegetable planting. We have developed a novel substrate solidified process with high-frequency electromagnetic heating, which significantly reduces energy consumption compared to the traditional curing process with steam heating. In this study, the effects of three modification methods (alkali modification, APTES modification, and alkali + APTES combined modification) on the physicochemical properties of jute were studied, and the strengthening effects of different modified jute fibers on solidification substrate were investigated. The results showed that the addition of jute fiber could improve the mechanical properties of the solidification substrate. Compared with the control group, the modified jute fiber could increase the breaking tension by 13.1~24.2 N, the impact toughness by 0.85~2.09 KJ/m^2^, and the hardness by 21.6~35.6 HA. Moreover, the addition of a small amount of jute fiber can effectively improve the mechanical properties and will not affect the growth of plant roots.

## 1. Introduction

Substrates are soilless cultivated materials that provide water, nutrients, and physical support for plants. Traditional soilless cultivation uses bulk substrates and requires the use of a large number of cultivation containers [1]. In addition, the water and gas characteristics of a traditional dispersed matrix completely depend on the ratio of substrate components, which is greatly affected by raw materials, and it is difficult to accurately regulate. In some special applications, such as the three-dimensional greening field, due to strong solar radiation and rain erosion, the traditional bulk substrate may lose more water and nutrients, resulting in high maintenance costs such as watering and fertilization [2]. Whether it is vertical greening facilities or roof greening facilities, the ordinary loose substrate cannot meet the fixation of water and nutrients [3]. Solidified forming substrate is a new type of substrate product which is bonded and solidified by substrate raw materials through a certain process, so that cultivation containers are not needed in cultivation and the influence of rain erosion can be reduced [4].

Compared with other soilless cultivation methods, solidified substrate culture has the advantage of diversified application scenarios. As a part of soilless culture, the significance of the existence of solidification substrate is not limited to the agricultural field, but also plays an important role in other fields. In daily life, the solidification substrate can be planted on the balcony to play a decorative role to achieve small-scale family planting. In the field of urban greening, the solidification substrate can realize roof greening and vertical greening, which can add greenery to the city and absorb greenhouse gases [5]. Solidification substrate products in the field of soilless cultivation represent an emerging industry with a broad consumer market. Therefore, the study of solidification substrate is of great significance to promote the development of modern agriculture and the social economy.

Common solidification substrate processes include the compression curing process, hydrogel curing process, foamed resin curing process, etc. The compression curing process is divided into hot compression and cold compression. Thermal compression is the method of heating compression, which softens the lignin in the biomass during the heating process and produces adhesion, making the substrate easier to cure and form [4]. Usually, the heating methods of thermal compression include steam heating, microwave heating, etc. These heating methods need to rise to a higher temperature to cure, which will lead to high energy consumption [6]. Cold compression directly applies pressure to the biomass solidification at room temperature. Cold-compression curing does not require heating, is more energy saving, and the process is the simplest. The compression of biomass at room temperature will generate a small amount of heat to soften lignin under the action of friction, which will provide a certain amount of help for bonding curing molding, but cold compression requires sufficient pressure for cure molding [7]. As a good hydrophilic and biocompatible material, hydrogels are widely used in the field of substrate cultivation [8]. Some researchers use hydrogels as substrates to preserve water and nutrients for seed growth [9]. In view of the requirements of plant roots for ventilation and water-holding pores, pore creation can effectively regulate the pore structure of hydrogel substrate and provide a suitable environment for plant growth [10]. However, with the increase in use time, the physical and chemical properties of hydrogel will change, including degradation and fragmentation. Foamed resin material has the characteristics of light texture, heat insulation, and easy degradation, so some researchers use it for cultivation [11], but the chemical properties of foamed resin itself may cause potential harm to the environment. In view of the current situation of single curing methods and the low efficiency of the substrate, using hot melt fiber as a binder to prepare new solidification substrate can effectively solve this problem. The raw material mixture is cured by special hot melt fibers at a certain temperature (about 130 °C). Unlike hydrogel substrate and resin foam solidification substrate, the hot melt fibers form a tightly connected three-dimensional fiber network in the solidification substrate, so that the solidification substrate can maintain a stable shape for a long time. Further compression curing makes the originally loose particles tightly connected and increases the number of small pores, thus enhancing the water absorption and retention capacity of the substrate. At the same time, increasing the connectivity of macropores contributes to the gas exchange between the interior and exterior of the substrate, increases the flow of water and the migration of solutes in the substrate, and gives the solidification substrate an excellent water and air permeability.

High-frequency heating technology is a non-contact heating method, the essence of which is that the conductor cuts the magnetic induction line to generate induced current and then generates heat from the thermal effect of the current [12]. High-frequency heating has the advantages of energy saving, environmental protection, no pollution, uniform heating, etc., and has been widely used in the industrial field. High-frequency heating technology is used to develop a device for preparing the solidification substrate, which can effectively reduce the energy consumption of the solidification substrate. High-frequency heating technology is used to heat the metal mold to a certain temperature to melt the hot melt fiber to the solid substrate, so as to reduce the production and processing cost of the solidification substrate and maximize the benefits of the solidification substrate products.

Due to the skin effect of high-frequency heating [13], heat is mainly concentrated outside the solidification substrate, and the heat inside the solidification substrate is insufficient. Therefore, such a heating method leads to the low adhesion of hot melt fibers inside the solidification substrate, and a new method to enhance the mechanical properties of the matrix is needed. In recent years, the surface treatment of hemp fiber has become a hot spot in the direction of reinforced polymer composites. Hemp fiber is rich in cellulose, hemicellulose, lignin, pectin, and other plant fiber-specific substances [14]. There are a large number of hydroxyl groups in the internal structure, which has hydrophilic properties. On the other hand, the thermoplastic hot melt fiber in the solidification substrate is a thermoplastic polymer. It has hydrophobic properties. There are obvious differences in surface properties between them. Therefore, in order to enable jute fiber to be better combined with hot melt fiber, and then enhance the mechanical properties of the solidification substrate, it is necessary to surface-treat the jute fiber. Existing research shows that jute fiber modification mainly includes physical and chemical methods: physical methods include steam blasting, heat treatment, and other methods; chemical methods include lye immersion, coupling agent encapsulation, graft modification, and other methods. In the research of removing surface impurities and hydrophobic modification, the use of lye immersion and coupling agent coating is more often used, so the method of alkali modification and coupling agent modification is used to treat the surface of jute fiber.

**Figure 1:**
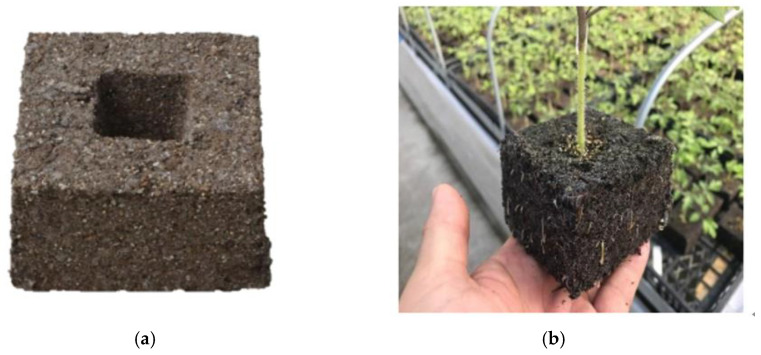
Solidification substrate molding effect: (**a**) solidification substrate; (**b**) cultivation effect of solidification substrate.

The solidification substrate in this study is composed of peat, vermiculite, perlite, and thermoplastic polymer hot melt fiber. By mixing the raw materials according to a specific proportion and placing them into the mold, the metal mold is heated rapidly by the principle of high-frequency heating. After reaching the melting softening point of the thermoplastic polymer hot melt fiber, the hot melt fiber will bond to other raw materials to solidify the substrate together and finally achieve the effect of solidifying the substrate. However, after melting and softening, the hot melt fibers in the solidification substrate will randomly bond to the nearby substrate raw materials, which makes the mechanical properties of the solidification substrate unstable. The hot melt fiber is a thermoplastic polymer fiber, and the main component contains a proportion of microplastics. Since the main application scenarios of the solidification substrate are vertical greening and roof greening, it does not come into direct contact with the soil and pollute the environment. On the other hand, the use of thermal melt fibers is only 5%, which is insignificant compared to the greening of plastic pot plants. As a kind of natural fiber, jute fiber will degrade naturally after being added to the solidification substrate and will not cause harm to the environment. Therefore, in this study, jute fiber was selected as the structural reinforcement material, and alkali treatment, silane coupling agent KH-550 treatment, and NaOH and KH-550 combined treatment were used to study the effects of different treatment methods on the mechanical properties of the solidification substrate. The structure and properties of jute fiber were characterized by SEM, XRD, TG, and FTIR. The solidification substrate was prepared by mixing jute fibers before and after treatment in cured matrix raw materials and then testing their mechanical properties. The presentation of the solidification substrate is shown in Figure 1.

## 2. Materials and Methods

### 2.1. Materials

The jute fiber used in this research comes from Changsha, Hunan, and the hot melt fiber (Hunan Shangjia Green Environment Co., Ltd. Changsha, China). Hot melt fiber is a kind of thermoplastic fiber which is composed of two component fibers with a skin core structure. The main components of hot melt fibers are polyester, polyamide, polyolefin, polyvinyl chloride, modified polyester, modified polyamide, or copolymers of the above polymers. The device used to prepare the solidification substrate is designed based on the principle of high-frequency heating. The main reagents used in the experiment are a 98% concentration of NaOH, a 95% concentration of KH-550, and a 5% concentration of acetic acid. The other raw materials used in the experiment were peat soil, vermiculite, and perlite. The peat (Pindstrup, Mariager, Denmark). Perlite and vermiculite were purchased (Weifang Youshun Environmental Protection Technology Co., Ltd. Shenzhen, China). All reagents used in this study were purchased (Shanghai McLean Biochemical Co., Ltd. Shanghai, China).

### 2.2. Jute Fiber Surface Treatment

Alkali treatment of jute fiber: First, sodium hydroxide and deionized water were mixed with a mass fraction of 5% sodium hydroxide solution. Then, the pre-dried jute fiber was soaked in sodium hydroxide solution for 1 H according to the mass ratio of 1:30, and the beaker mouth was sealed with plastic wrap. Finally, the jute fiber was washed with 5% acetic acid solution to a neutral pH, and then put into an 80 °C electric blast drying oven for 24 h. After the jute fiber was soaked in various liquids, the water used for soaking constituted a harmless treatment.

Silane coupling agent treatment of jute fiber: Firstly, ethanol solution with mass fraction ratio of 2:3 was prepared with anhydrous ethanol and deionized water. Then, the silane coupling agent KH-550 solution with a mass ratio of 5% was poured into the configured ethanol solution and quickly stirred evenly, and the pH value was adjusted to neutral with a concentration of 5% acetic acid solution. Then, the dried jute fiber was soaked in the solution at the ratio of 1:30 for 1 h, and the beaker mouth was sealed with plastic wrap during the soaking process. Finally, the jute fibers were taken out and dried in an electric blast drying oven at 80 °C for 24 h.

Combined treatment of jute fiber: the alkali-treated jute fiber was dried and then treated repeatedly using the silane coupling agent to achieve the combined treatment of jute fiber.

### 2.3. Preparation of Jute Fiber-Reinforced Solidification Substrate

**Figure 2:**
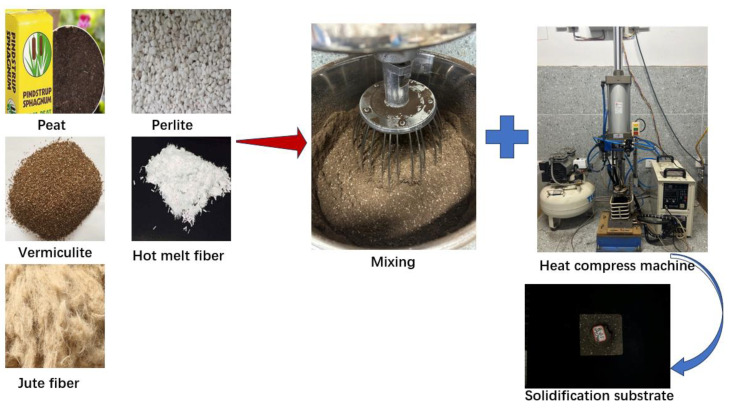
Flow chart of solidification substrate preparation.

According to the volume ratio of 9:3:1, peat, perlite, and vermiculite are configured as the base part of the solidification substrate, and the volume of the base part accounts for 95% of the total formula. Then, we add the jute fiber into the blender and mix at a constant speed for 30 min. After stirring, hot melt fibers with a volume ratio of 5% are added and stirring continued for 30 min until the hot melt fibers are evenly distributed in the matrix. We weigh 130 g solidification substrate raw materials into a beaker and pack it into a 9 × 9 × 10 cm^3^ cast iron profile mold for compaction, where the inner wall of the mold is 3 mm thick. Then, we start the air compressor and raise the compression lever to adjust the compression ratio to 0.6. The meaning of 0.6 compression ratio is the value obtained by dividing the height of the solidification substrate by the height of the mold. Next, we start the air compressor, and the compressed air will reach the air pressure device through the plastic pipe, so that the mold pressure plate has the power of compression. Then, we start the high-frequency heating device, energize the water pump to move the cooling water through the copper tube uniform cooling coil, and set the heating current to 10 A. When the temperature of the top surface of the mold is 200 °C, the heating is stopped and the light is turned on by pressing the pressure device to press the top surface of the mold into the mold for 30 s. In addition, when the temperature of the mold reaches 200 °C, the energy consumption is about 0.06 KWH. When the pressure holding time is over, we loosen the pressure device switch and remove the solidification substrate. The specific process is shown in Figure 2.

### 2.4. Characterization of Jute Fibers

Jute fibers were observed with a stereomicroscope (SJ-500; Suzhou Shenying Optical Co., Ltd., Suzhou, China) To observe the fiber diameter change, we took the jute fiber sample before and after surface treatment and placed it on the loading platform to adjust the light intensity. The objective magnification was observed. Observation conditions: eyepiece multiplier 10×; objective multiplier depends on the observation. SEM (GeminiSEM360; Carl Zeiss AG, Oberkochen, Germany) was used to observe the surface morphology, After a small amount of sample was sprayed in the gold spraying furnace, the samples were glued to the sample table with conductive adhesive to observe the surface morphology. An X-ray diffraction analyzer (Uitima IV; RigaKu, Ann Arbor, MI, USA) was used for the calculation of crystallinity; the sample is ground into powder and evenly dispersed on the slide. The test conditions were the conventional mode (5~90°), a scanning rate of 5°/min, and a test angle range of 10~90°. Formula (1) was used to calculate the crystallinity of the sample. The crystallinity calculation formula is shown in Formula (1). FTIR (Spectrum3; PerkinElmer, Waltham, MA, USA) was used to test the surface functional groups. The test conditions are a resolution of 4 cm^−^^1^, a frequency range of 650–4000 cm^−^^1^, and the number of scans is 32. A thermogravimetric analyzer (TGA 8000; PerkinElmer) test was used for quality loss. After the samples were ground by a ball mill, 3–5 mg of debris was placed in the alumina crucible, and the temperature rise rate from room temperature to 500 °C at 10 °C/min under nitrogen conditions was recorded.

(1) $$X_{c}=\frac{{\Sigma I}_{c}}{\Sigma I_{c}+{\Sigma I}_{a}}\times 100\%$$

where ${\Sigma I}_{C}$ is the diffraction peak intensity of the crystalline part and ${\Sigma I}_{a}$ is the diffraction peak intensity of the non-crystalline part.

### 2.5. Mechanical Properties Test of Solidification Substrate

Due to the strengthening effect of jute fiber, it was necessary to carry out a series of mechanical properties tests on the prepared jute fiber. We tested the tensile force at break with a Push tensile testing machine (DS2; Shenzhen Hongyuan Electronic Instrument Co., Ltd., Shenzhen, China). We tested the impact toughness with a pendulum impact testing machine (250-J; Jinan Times Testing Instrument Co., Ltd., Jinan, China) We tested the surface hardness with a Shore hardness tester (HLX-AC; Nanjing Su Measuring Instrument Co., Ltd., Nanjing, China).

### 2.6. Statistical Analysis

All statistical analyses were performed using Ermolin et al.’s [15] method and EXCEL. All graphs were drawn using Origin2024, and all data in this article are averages of three repeated measurements.

## 3. Results and Discussion

### 3.1. Influence of Surface Treatment on the Composition and Structure of Jute Fiber

#### 3.1.1. Fiber Diameter

The diameter changes in jute fiber before and after modification were observed by stereomicroscope, as shown in Table 1, using the measuring software that comes with the asanas microscope to measure the diameter of the jute fibers. Since the fiber is actually elliptical, for the purpose of measuring the diameter, in this study, it is assumed that the cross-section of the jute fiber is circular. It can be seen from the table that after the alkali modification of jute fiber, the fiber diameter decreased from 28.3 μm before treatment to 23.9 μm after. Combined with FTIR analysis, it can be obtained that the surface hemicellulose and lignin and other impurities of jute fiber are removed after soaking in sodium hydroxide solution, resulting in a decrease in fiber diameter. After modification by KH-550, the diameter of jute fiber increased slightly, which may be caused by the silane coupling agent attached to the fiber surface. The diameter of jute fiber modified by NaOH and KH-550 was slightly increased compared with alkali treatment [16].

#### 3.1.2. Surface Topography

**Figure 3:**
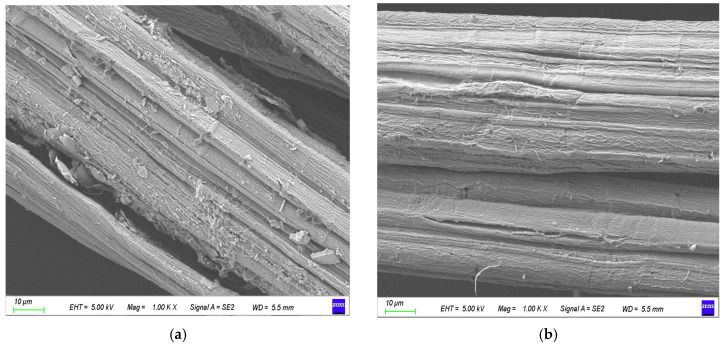

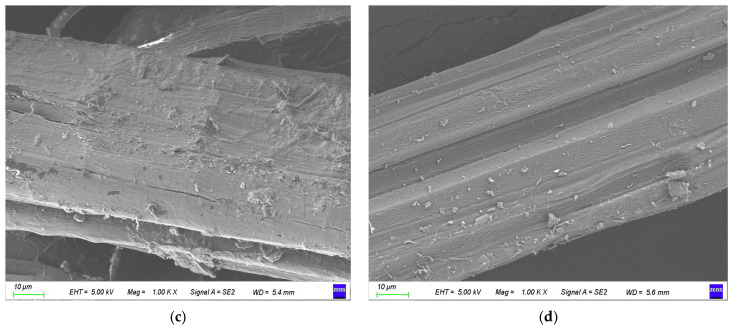
Surface morphology of jute fiber before and after modification. (**a**) Unmodified, (**b**) NaOH modification, (**c**) KH-550 modification, and (**d**) combined modification by NaOH and KH-550.

A scanning electron microscope was used to observe the changes in surface morphology of jute fiber before and after modification. The main components of jute fiber are cellulose, hemicellulose, and lignin. Cellulose is made from glucose molecules linked by a β-1, 4-glucoside bond. Glucose is a six-carbon sugar, and multiple glucose molecules are connected to form long chains of macromolecules. Multiple molecular chains intertwine and aggregate to further form microfilaments, which can be assembled into larger fiber structures. This multi-level structure gives high strength and stability to cellulose, which is the main component of the plant cell wall and plays a role in supporting and protecting plant cells. Figure 3 is the SEM image of jute fiber after 1000-times magnification before and after modification. As can be seen from the figure, there are many impurities on the surface of unmodified jute fiber, which are hemicellulose, lignin, pectin, and other phenomena attached to the surface of cellulose [17]. The presence of impurities and defects will adversely affect the interfacial bonding of composites. NaOH-modified jute fiber can effectively remove small impurities such as hemicellulose and lignin, which verifies the conclusion that the diameter of the fiber bundle becomes smaller.

The surface texture of jute fiber can be clearly seen after NaOH modification, which can increase the surface area of the fiber. At the same time, the impurities on the surface of jute fiber have been basically removed and the fiber has clear lines, which can enhance the interface bonding force of the composite material to a certain extent. As can be seen from Figure 3c, the surface of the jute fiber is coated with a layer of KH-550, which makes the jute fiber more hydrophobic. Jute fiber was modified by KH-550, which caused a chemical reaction on the surface of jute fiber and formed chemical bonds. The silane coupling agent evenly coating the jute fiber was beneficial to better bond with the hot melt fiber [18]. NaOH and KH-550 combined modified jute fiber to remove the impurities on the surface of the fiber to make the surface of the fiber rough, and KH-550 could better wrap the jute fiber. The increase in roughness can increase the mechanical interlocking ability between jute fiber and hot melt fiber matrix, and KH-550 can provide a better bonding interface [19]. The reaction between KH-550 and jute fiber is shown in Formula (2).
(2)
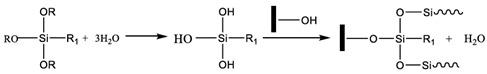



#### 3.1.3. Chemical Structure

**Figure 4:**
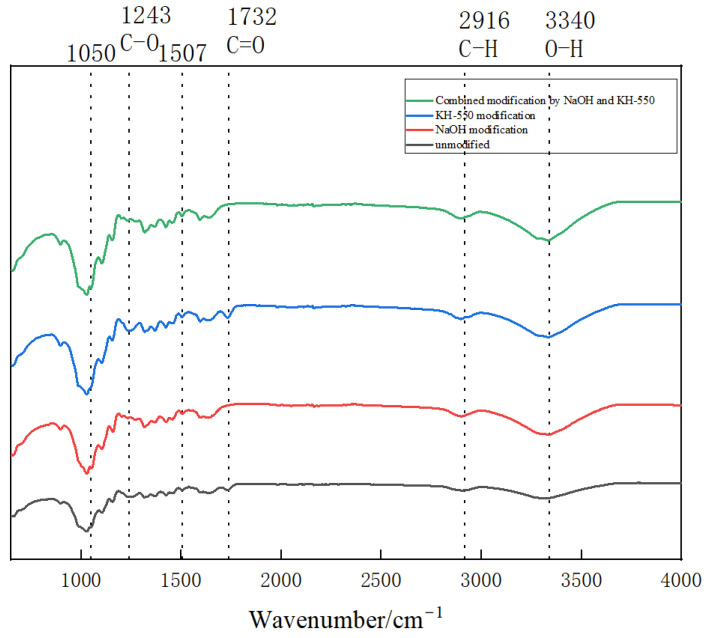
FTIR spectra of jute fiber before and after modification.

The changes in the surface composition of jute fiber before and after surface treatment were characterized by FTIR. Figure 4 shows the infrared spectra of jute fiber before and after modification, from which it can be seen that the absorption peak at 3340 cm^−1^ belongs to hydroxyl vibration. The results indicated that the cellulose crystal zone and the hydrogen bond network formed by jute fiber were destroyed after alkali treatment, and the hydroxyl vibration peak became wider. The vibration of the C-H bond in 2916 cm^−1^ attributed to cellulose indicates that a low concentration of sodium hydroxide can open the crystalline zone of jute fiber and cause the vibration of C-H bond strength improvement [20]. The 1732 cm^−1^ absorption peak is the C=O vibration peak attributed to hemicellulose, and the 1243 cm^−1^ absorption peak is the C-O vibration peak attributed to lignin [21]. After alkali modification, these two vibration peaks of jute fiber were obviously weakened. In addition, the absorption peak at 1507 cm^−1^ belonged to the characteristic vibration peak of the lignin benzene ring skeleton, which was significantly reduced after alkali modification. The infrared spectrum of jute fiber modified with silane coupling agent has no obvious change. The absorption peak at 1050 cm^−1^ is derived from the Si-O vibration peak in the silane coupling agent, which can be seen as slightly enhanced in the figure. The reason of no obvious difference may be that the characteristic peak of jute fiber overlaps with that of silane coupling agent Si-O. The absorption peak of jute fiber modified by coupling agent at 1243 cm^−1^ is stronger than that of unmodified jute fiber, which may be caused by the -Si-O-C- bond between the silane coupling agent and the hydroxyl group on the surface of jute fiber after hydrolysis.

### 3.2. Effect of Surface Treatment on Thermal Properties of Jute Fiber

**Figure 5:**
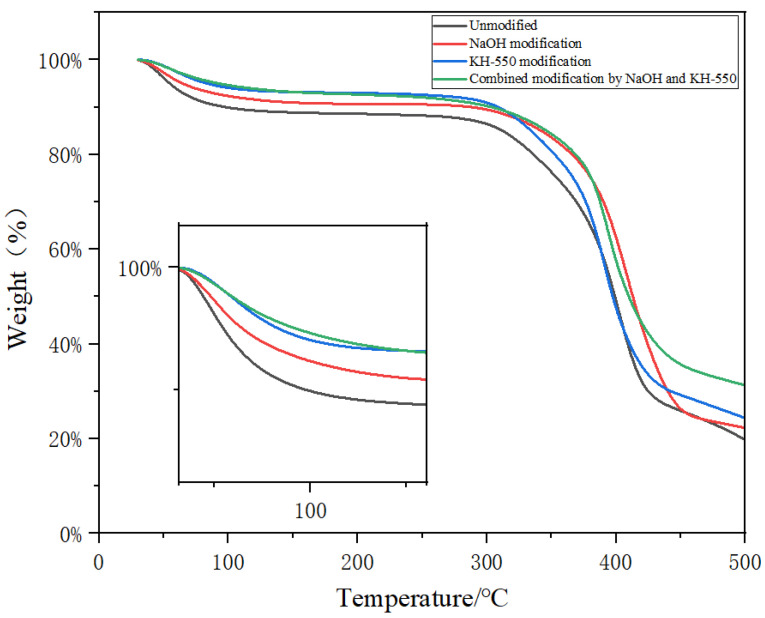
TGA curves of jute fiber before and after modification.

A thermogravimetric analyzer was used to characterize the thermal stability of jute fiber before and after modification. Figure 5 shows the thermogravimetric curve of jute fiber before and after modification. As can be seen from the figure, the thermogravimetric curve of jute fiber before and after modification is similar, and after drying with water loss during the initial heating process, a small weight-loss phenomenon occurs at about 90 °C. This is because jute fibers contain a small amount of water, which evaporates during the heating process resulting in weight loss. The difference is that the heat loss of modified jute fiber is less than that of unmodified jute fiber. This is because the dehydration reaction occurs when the alkali-modified jute fiber, removing the hemicellulose, lignin, and water together, and the silane coupling agent react with the hydroxyl group on the surface of the jute fiber, thereby reducing the absorption of water. The decomposition temperature of hemicellulose and cellulose in jute fiber is 200~350 °C, at which time hemicellulose and cellulose are basically completely decomposed [22]. When the temperature rises to 500 °C, the thermogravimetric curve is basically stable, indicating that the jute fiber is completely decomposed. Table 2 shows the temperature values required by different modification methods when the weight loss of jute fiber is 5% and 10%. It can be seen from the table that the thermal decomposition temperature of modified jute fiber is higher, and the combined modification of alkali and silane coupling agent has a better thermal stability than other jute fibers.

### 3.3. Influence of Surface Treatment on Crystallinity of Jute Fiber

**Figure 6:**
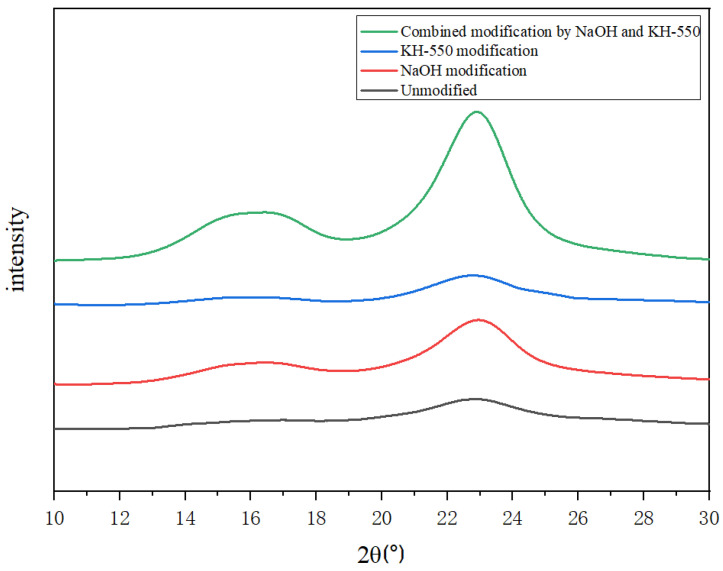
XRD pattern of jute fiber before and after modification.

The crystal structure and crystallinity of jute fiber before and after modification were measured by an X-ray diffraction analyzer. Figure 6 is the XRD pattern of jute fiber before and after modification. It can be seen from the figure that the (101) crystal plane and (002) crystal plane corresponding to the jute fiber appear as diffraction peaks when 2θ = 16.06° and 22.7° [23]. This is a typical cellulose class I crystal type. The results show that the three modification methods have no effect on the crystal shape of jute fiber crystals. There are both crystalline and non-crystalline regions in jute cellulose, which are gradually transitioned without specific regions. In jute fiber after soaking in sodium hydroxide to remove most of the lignin and hemicellulose, the proportion of cellulose in jute fiber was increased. The low concentration of sodium hydroxide solution mainly acts on the amorphous region of jute fiber, so that the lignin and the hydroxyl group in the hemicellulose in this region are more exposed. These hydroxyl groups are combined with the crystalline zone of jute fiber through hydrogen bonding, resulting in orientation, which further improves the crystallinity of jute fiber. The crystallinity change in jute fiber before and after modification obtained by the crystallinity formula is shown in Table 3. It can be seen from the table that the crystallinity of unmodified jute fiber is 41.66%. After alkali modification, the fiber crystallinity increased to 45.09%, which was caused by the removal of hemicellulose and lignin in jute fiber. The crystallinity of jute fiber modified by the silane coupling agent decreased to 34.01%, because the reaction between the silane coupling agent and cellulose caused an increase in amorphous cellulose after hydrolysis. The reaction mainly occurred in the amorphous region and the edge of the crystalline region of the cellulose. This may break the hydrogen bonds between the cellulose chains and allow silane coupling agents to penetrate between the cellulose molecular chains, further reducing the crystallinity of the cellulose. In the combined treatment, a low concentration of sodium hydroxide is first used to remove the components of the amorphous area on the surface of the jute fiber, so that the cellulose molecular chains are arranged more neatly. Then silane coupling agent is used to wrap jute fiber to restrict the movement of molecular chains, so that the molecular chains which have been close to regular arrangement can be further fixed in the restricted state. This modification treatment stabilizes and strengthens the crystal structure of jute fiber and greatly improves the crystallinity of jute fiber.

### 3.4. Influence of Surface Treatment on the Interface Properties of Structure-Enhanced Solidification Substrate

**Figure 7:**
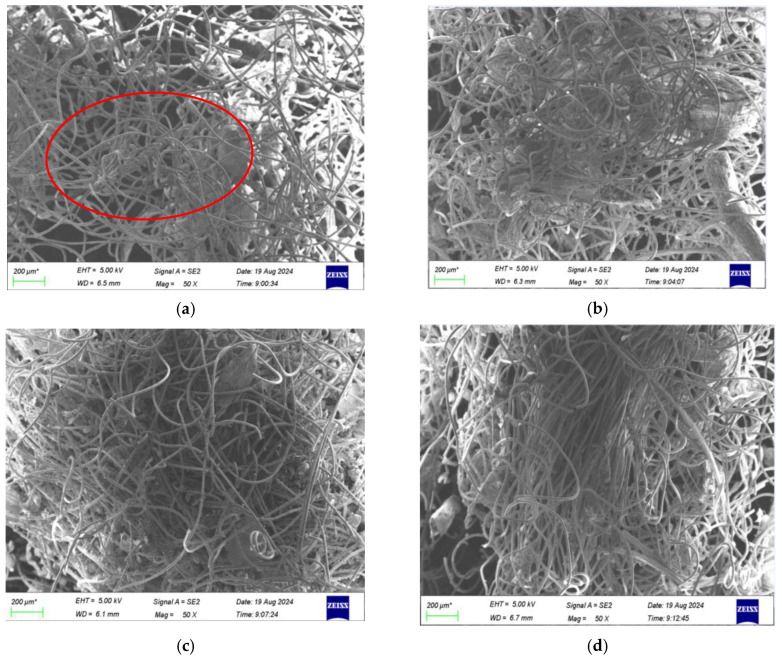
SEM images of the inside of the solidification substrate before and after the modification of jute fiber. (**a**) Unmodified, (**b**) NaOH modification, (**c**) KH-550 modification, and (**d**) combined modification by NaOH and KH-550.

Figure 7 shows the SEM images inside the solidification substrate prepared by adding jute fibers of different treatments. In Figure 7a, there are obvious holes at the interface of the jute fiber as a structural reinforcement fiber and the hot melt fiber matrix, and only partially melted or softened hot melt fiber is bonded in a disorderly manner on the surface of unmodified jute fiber. The hot melt fiber cannot completely wrap the unmodified jute fiber, because there are pectin and wax layers on the surface of the unmodified jute fiber, which cannot be well combined with the hot melt fiber, as shown in Figure 7b. The alkali-modified jute fiber was fully covered by the hot melt fiber, but there were still some small holes. The results show that the surface of jute fiber can be roughened by removing part of hemicellulose, lignin, and impurities with alkali, and the interface between jute fiber and hot melt fiber can be mechanically interlocked to improve the strength of the solidification substrate. On the other hand, low-concentration sodium hydroxide solution can enhance the activation energy of the cellulose hydroxyl group, break hydrogen bonds, change the local structure of the jute fiber surface, and make jute fiber more hydrophobic. Thus, it can be better combined with hot melt fibers. As shown in Figure 7c,d, hot melt fibers can fully wrap jute fibers, indicating that the silane coupling agent can improve the interface bonding between jute fibers and the hot melt fiber matrix. After modification by the silane coupling agent, the coupling agent is immersed into the jute fiber, and the hydroxyl group of jute fiber is replaced by the coupling agent, which reduces the hydrophilicity of the jute fiber and improves the interface bonding ability of the jute fiber.

### 3.5. Effect of Surface Treatment on Mechanical Properties of Solidification Substrate with Structural Reinforced Fiber

**Figure 8:**
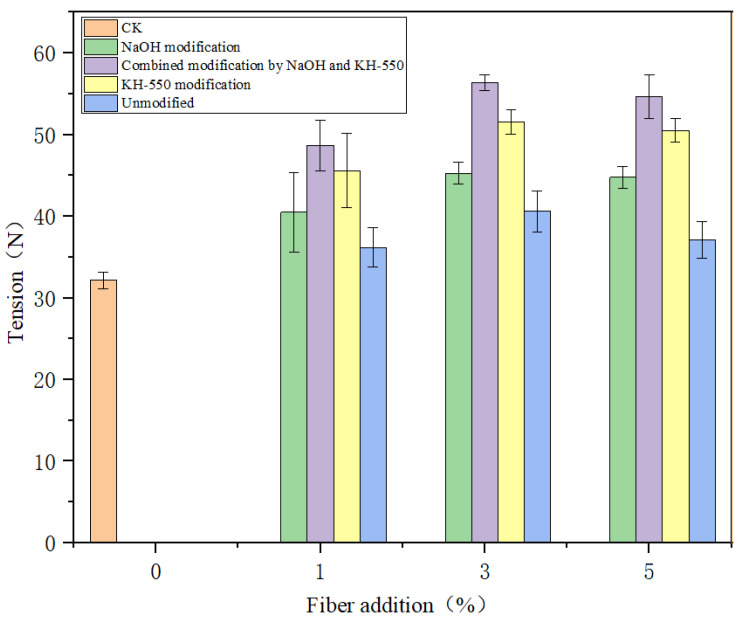
Tension of jute fiber solidification substrate.

The solidification substrate was prepared by adding jute fiber into the solidification substrate raw material and then placed in a ventilated place to air-dry to a constant weight to test the mechanical properties of the solidification substrate. CK (solidification substrate without jute fiber added) was selected as the control group, and the solidification substrate prepared by adding unmodified jute fiber, alkali-modified jute fiber, KH-550-modified jute fiber, and combined modified jute fiber was produced according to different proportions for tensile testing. Figure 8 shows the comparison of the fracture tensile properties of different solidification substrate prepared before and after the modification of jute fiber. Different fiber addition amounts (1%, 3%, and 5%) were controlled to screen out the optimal fiber addition amount. It can be seen from the figure that the solidification substrate has the best mechanical properties when the fiber content is 3%. When no jute fiber is added, the tension of the solidification substrate is completely conducted between the hot melt fibers. Because there is no jute fiber as a structural reinforcement material, the tension conduction to the hot melt fiber is not well bonded to the convenient fracture. Therefore, the mechanical properties of the control group were the worst, only 32.1 N. The less jute fiber added, the less jute fiber can be combined with the hot melt fiber. This makes the hot melt fiber more combined with peat, vermiculite and perlite components, which reduces the mechanical properties of the solidification substrate. When the content of jute fiber is 5%, there is too much fiber in the mixing process of the matrix raw materials, which leads to the fiber winding on the mixer, resulting in uneven mixing. Therefore, the mechanical properties of the solidification substrate with the addition of 5% jute fiber will decrease. In terms of modification methods, the three modification methods can effectively enhance the mechanical properties of the solidification substrate. Compared with the unmodified jute fiber solidification substrate, the fracture tension of the combined modified solidification substrate can be increased by 51.7~76.3%. Alkali soaking makes the surface of jute fiber rough and provides a good reaction site for the silane coupling agent. The -NH_2_ in the silane coupling agent can bind with the groups at the end of the hot melt fiber and improve the interface bonding strength between jute fiber and hot melt fiber. Because of the good mechanical interlocking between the fiber and the matrix, and the strong chemical bond cooperation, the jute fiber is evenly and regularly distributed in the solidification substrate. Therefore, the solidification substrate prepared by the combined modification of jute fiber has the best mechanical properties.

### 3.6. Influence of Surface Treatment on Impact Toughness of Solidification Substrate

**Figure 9:**
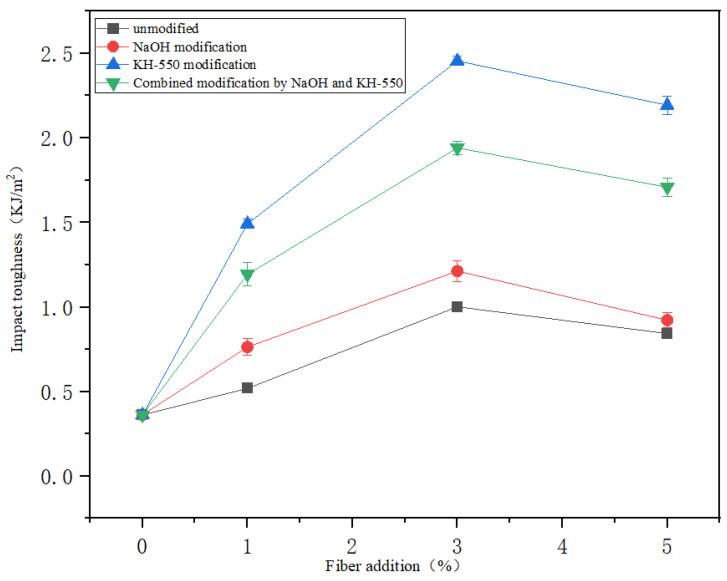
Impact toughness of jute fiber solidification substrate.

The impact toughness of the solidification substrate was tested with a pendulum impact tester to simulate the ability of the solidification substrate to resist damage under impact in the actual application scenario. Due to the high strength and toughness of jute fiber itself, when added to the solidification substrate, jute fiber can act as a skeleton to bear and disperse the impact force. Upon impact, the impact force is transmitted through the substrate to the jute fibers, which absorb and consume energy, thus resisting deformation and breakage. After the solidification substrate of jute fiber was prepared, it was naturally air-dried to a constant weight and then placed on the impact test machine to test its impact toughness. Figure 9 shows the impact toughness test results of the jute fiber solidification substrate. It can be seen from the figure that adding jute fiber can improve the impact toughness of the solidification substrate. Without jute fiber, the impact force of the solidification substrate cannot be effectively transmitted, and the impact toughness is only 0.36 KJ/m^2^. The impact toughness of solidification substrate is related to the combination of jute fiber and hot melt fiber. Among all the addition methods, the impact toughness of jute fiber solidification substrate modified by a coupling agent is the strongest, which is 0.97~1.45 KJ/m^2^ higher than that of the unmodified jute fiber solidification substrate. It shows that jute fiber modified by the coupling agent can fully disperse the impact force. Although alkali modification can improve the roughness of the fiber surface, it may lead to increased incompatibility between the fiber surface and the hot melt fiber matrix, and a weak point is formed at the interface joint. At the same time, the concentration of lye may be higher, which may cause the strength of jute fiber itself to decrease, so the effect of alkali modification and combined modification to enhance the impact toughness is lower than that of coupling agent modification.

### 3.7. Influence of Surface Treatment on Hardness of Solidification Substrate

When the jute fiber before and after surface treatment is added into the solidification substrate, the hardness of the substrate will also be improved while the mechanical properties are improved. Jute fiber itself has a certain stiffness; adding jute fiber before and after surface treatment into the solidification substrate can effectively conduct stress and limit the deformation ability of the solidification substrate, thus increasing the hardness. On the other hand, there is a better interface between the modified jute fiber and the hot melt fiber, which reduces the interface defects and stress concentration. When the solidification substrate is subjected to external forces, it can better cooperate with the force. However, the good bonding interface makes the relative displacement of each component in the solidification substrate difficult, which leads to an increase in substrate hardness. The increase in substrate hardness makes it difficult for plants to take root. In the early stage of plant growth, root growth will be subjected to greater mechanical resistance, and it is difficult to penetrate the substrate. In severe cases, root growth will be limited and the root will become shorter and thicker.

**Figure 10:**
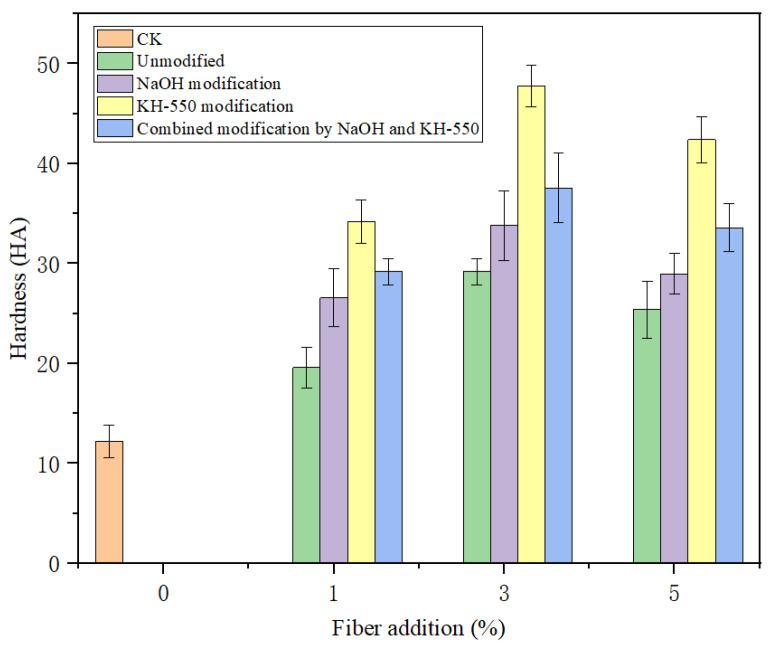
Hardness of jute fiber solidification substrate.

**Figure 11:**
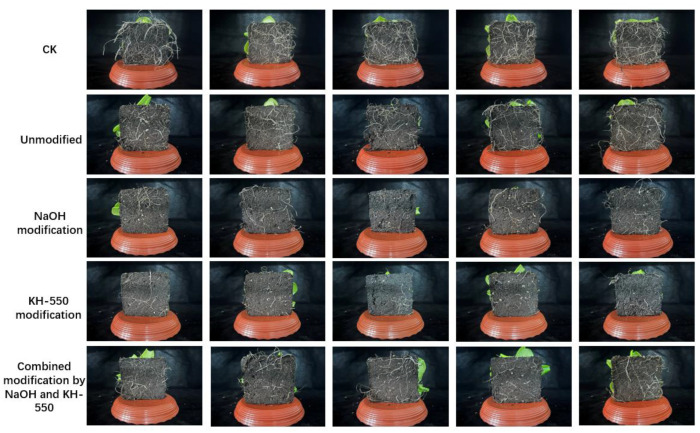
Effect of jute fiber solidification substrate planting.

The hardness changes in the jute fiber solidification substrate before and after modification are shown in Figure 10. It can be seen from the figure that with the increase in jute fiber addition, the hardness of the cured matrix showed a different upward trend. The hardness of unmodified jute fiber solidification substrate increased gently, between 19.6 and 29.2 HA. The hardness of jute fiber solidification substrate modified by the coupling agent increased significantly, ranging from 34.2 HA to 47.8 HA. The results show that adding the coupling agent to modify jute fiber can make the components in the solidification substrate bond more tightly and increase the substrate hardness. The reason for the decrease in hardness in the treatment of jute fiber addition of 5% is that the fiber is wrapped around the agitator during the stirring process and cannot be evenly distributed in the solidification substrate raw materials. In general, with the increase in jute fiber addition, the hardness of all cured substrates showed an upward trend. Different modification methods have different effects on the hardness enhancement of a cured matrix. Figure 11 shows the root growth of the jute fiber curing substrate in the early stage of cultivation.

The effect of jute fiber solidification substrate on plant root growth is shown in Figure 11. It was found that the hardness of the solidification substrate with 3% jute fiber was closely related to plant growth. On the seventh day of cultivation, it was found that because the hardness of the control group was the lowest, the lettuce roots could be smoothly extended, branched, and fixed, so the growth of the control group was the best. The growth rate of lettuce cured with unmodified jute fiber and alkali-modified jute fiber was slightly lower than that of the control group. When the hardness of the solidification substrate exceeds 35 HA, the lettuce root growth will be subjected to greater mechanical resistance, making it difficult for the root system to penetrate and extend outside the substrate, thus affecting the growth of the above-ground part.

## 4. Conclusions

(1) The mechanical properties of solidification substrate can be improved by alkali modification, silane coupling agent modification, and combined modification of jute fiber. It was found that alkali-modified jute fiber can remove impurities such as hemicellulose and lignin on the surface of the fiber and make the surface of the jute fiber rough, reduce its diameter, and increase its crystallinity. The silane coupling agent-modified jute fiber can wrap a layer of coupling agent molecules onto the surface of the fiber and enhance the hydrophobicity of the jute fiber. The modified jute fiber can increase the thermal stability and change the crystallinity of cellulose.
(2) The jute fibers before and after modification were prepared into different types of curing substrates. Through SEM characterization of the inside of the solidification substrate, it can be seen that the three modification methods have an obvious effect on improving the mechanical properties of the solidification substrate. The combined modification of jute fiber has the most obvious effect on the fracture tensile strength of the solidification substrate. The modification of the coupling agent improved the impact toughness of the solidification substrate the most obviously, and the coupling agent also increased the hardness of the solidification substrate, which had a certain effect on the growth of plant roots. In summary, the combined modification of jute fiber is the most beneficial to improve the mechanical properties of the solidification substrate.
(3) The addition of hot melt fibers as binders to solidification substrate producing microplastics has been a concern in the current study. However, considering that in actual production and application scenarios, the amount of microplastics generated by the solidification substrate is extremely limited and does not come into direct contact with the soil, the potential harm to the environment is not large. Compared to the amount of plastic used in potted plants, the amount of microplastics produced by solidification substrate is minuscule. The solidification substrate can be reused throughout the growth cycle of the plant, and can be replaced with a new solidification substrate immediately after the end of the cured substrate use cycle. The replaced solidification substrate can be converted into fertilizer by compost fermentation to provide nutrients needed for plant growth.

## Figures and Tables

**Table 1:** Changes in jute fiber diameter before and after modification.

Surface Treatment Mode	Jute Fiber Diameter (μm)
Unmodified	28.3
NaOH modification	23.9
KH-550 modification	31.4
Combined modification by NaOH and KH-550	35.6

**Table 2:** Temperature comparison of jute fiber before and after modification with the same weight-loss rate.

Surface Treatment Mode	Temperature (°C)	
	Weightlessness 5%	Weightlessness 10%
Unmodified	53.3	97.6
NaOH modification	64.3	148
KH-550 modification	83.3	303.6
Combined modification by NaOH and KH-550	92.6	308.3

**Table 3:** Comparison of crystallinity changes in jute fiber before and after modification.

Jute Fiber Modification Method	$X_{C}$ (%)
Unmodified	41.66
NaOH modification	45.09
KH-550 modification	34.01
Combined modification by NaOH and KH-550	52.69

## Data Availability

The original contributions presented in this study are included in the article. Further inquiries can be directed to the corresponding authors.
